# An immunogenomic signature for molecular classification in hepatocellular carcinoma

**DOI:** 10.1016/j.omtn.2021.06.024

**Published:** 2021-07-02

**Authors:** Weiwei Zhuang, Hongwei Sun, Shanshan Zhang, Yilin Zhou, Wanqing Weng, Boda Wu, Tingbo Ye, Weiguo Huang, Zhuo Lin, Liang Shi, Keqing Shi

**Affiliations:** 1 Translational Medicine Laboratory, The First Affiliated Hospital of Wenzhou Medical University, Wenzhou 325015, Zhejiang Province, P.R. China; 2 Department of Hepatobiliary Surgery, The First Affiliated Hospital of Wenzhou Medical University, Wenzhou 325015, Zhejiang Province, P.R. China; 3 The State University of New York at Stony Brook, NY, USA; 4 Department of Liver Diseases, The First Affiliated Hospital of Wenzhou Medical University, Wenzhou 325015, Zhejiang Province, P.R. China; 5 Department of Clinical Laboratory Medicine, The Eighth Affiliated Hospital, Sun Yat-sen University, Shenzhen 518033, Guangdong Province, P.R. China

**Keywords:** hepatocellular carcinoma, immunity, classification, overall survival, risk score

## Abstract

Immunity plays an important role in tumor development. In this study, we aimed to investigate molecular classification and its prognostic value in hepatocellular carcinoma (HCC) based on immune signature. Gene set enrichment analysis (GSEA) was used to calculate scores of immune pathways for HCC and hierarchical clustering in two databases (The Cancer Genome Atlas [TCGA], Liver Cancer-RIKEN, JP [LIRI_JP]). The scores of the immune microenvironment and the proportions of 22 immune cells were also calculated. Single-sample GSEA (ssGSEA) was used to screen survival prognosis-related immune pathways and calculate the hazard radio of differentially expressed immune-related genes (IRGs), which were validated in clinical samples and multiple datasets. Based on the immune characteristics, we identified three HCC subtypes, namely immunity high (Immunity_H), immunity medium (Immunity_M), and immunity low (Immunity_L), and confirmed that the classification was reliable and predictable. Immunity_H with a higher immune and stromal score indicated better survival rate. Cox regression analysis showed that *IL18RAP* and *IL7R* were the protective genes. Immune risk score was the independent risk factor of overall survival in HCC patients. These results indicated that immunogenomic classification could distinguish HCC patients with different immune status, which could impact the prognosis of the patients with HCC.

## Graphical abstract

**Figure:**
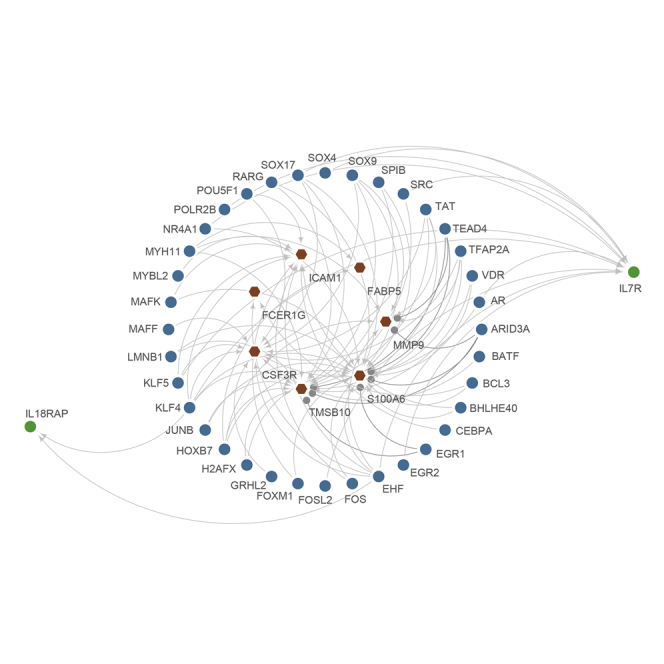

## Teaser

Weiwei et al. provided an immunogenomic classification based on immune characteristics to distinguish hepatocellular carcinoma (HCC) patients with different immune status, and the prediction model can be used for individual immunotherapy of HCC patients.

## Introduction

Hepatocellular carcinoma (HCC) is a common cancer worldwide with a high mortality rate.^1^^,^^2^ The pathogenesis of HCC is a complex process, which is influenced by multiple factors, such as environmental factors and the individual’s own genes.^3^ A large number of previous studies have indicated that the immune microenvironment of the primary tumor is an important prognostic factor.^4^ However, effective diagnostic indicators of the immune microenvironment are still lacking, resulting in fuzzy prognosis accuracy in HCC. The conventional treatments of HCC include surgery, chemotherapy, and radiotherapy,^5^^,^^6^ which have a better curative effect in the early stages of cancer. Therefore, it is urgent to find biomarkers for early detection and prognostic evaluation of HCC.^2^

The immunogenomic classification will help to guide the differential and effective treatment of HCC in an early stage and improve the accuracy of prognosis evaluation. Tumor-associated immune response plays an important role in cancer pathogenesis.^7^ Several cellular phenomena such as alterations in tumor microenvironment, inflammation, oxidative stress, and hypoxia facilitate tumor initiation, progression, and metastasis.^8^ T cells and natural killer (NK) cells play the role of immune surveillance. NK cells have strong anti-tumor activity and release perforin/granzymes or activate apoptosis pathways to kill tumor cells.^9^ In addition, NK cells can also secrete cytokines, such as interferon (IFN)-γ and tumor necrosis factor (TNF)-α, to inhibit tumor cell proliferation, tumor angiogenesis, and multistage canceration.^10^ Macrophages promote cell proliferation, infiltration, and tumor neovascularization. In addition, cancer immunotherapy as an innovative treatment method has become a hotspot in the field of cancer therapy research. At present, many cytokines, such as TNF, IFN-γ, and interleukin (IL)-2, have been correlated with the response of HCC immunotherapy.^7^ However, there are still many difficulties and problems in immunotherapy against HCC, such as the inability to evaluate the immunotherapy effect in advance.

In this study, we analyzed immunogenomic profiling of HCC patients and classified them into three different subtypes: immunity high (Immunity_H), immunity medium (Immunity_M), and immunity low (Immunity_L). We focused on analyzing two independent datasets of HCC, proving the reliability and reproducibility of this classification. Moreover, we identified the subtype-specific molecular features, including networks, pathways, genes, and gene ontology, according to the immune subtypes, and risk score was used to predict survival in patients with HCC. The identification of HCC subtypes associated with immune related genes would be advantageous for HCC patients who have responded to immunotherapy.

## Results

### Immune-related pathways profiling identified three HCC clusters

**Figure 1:**
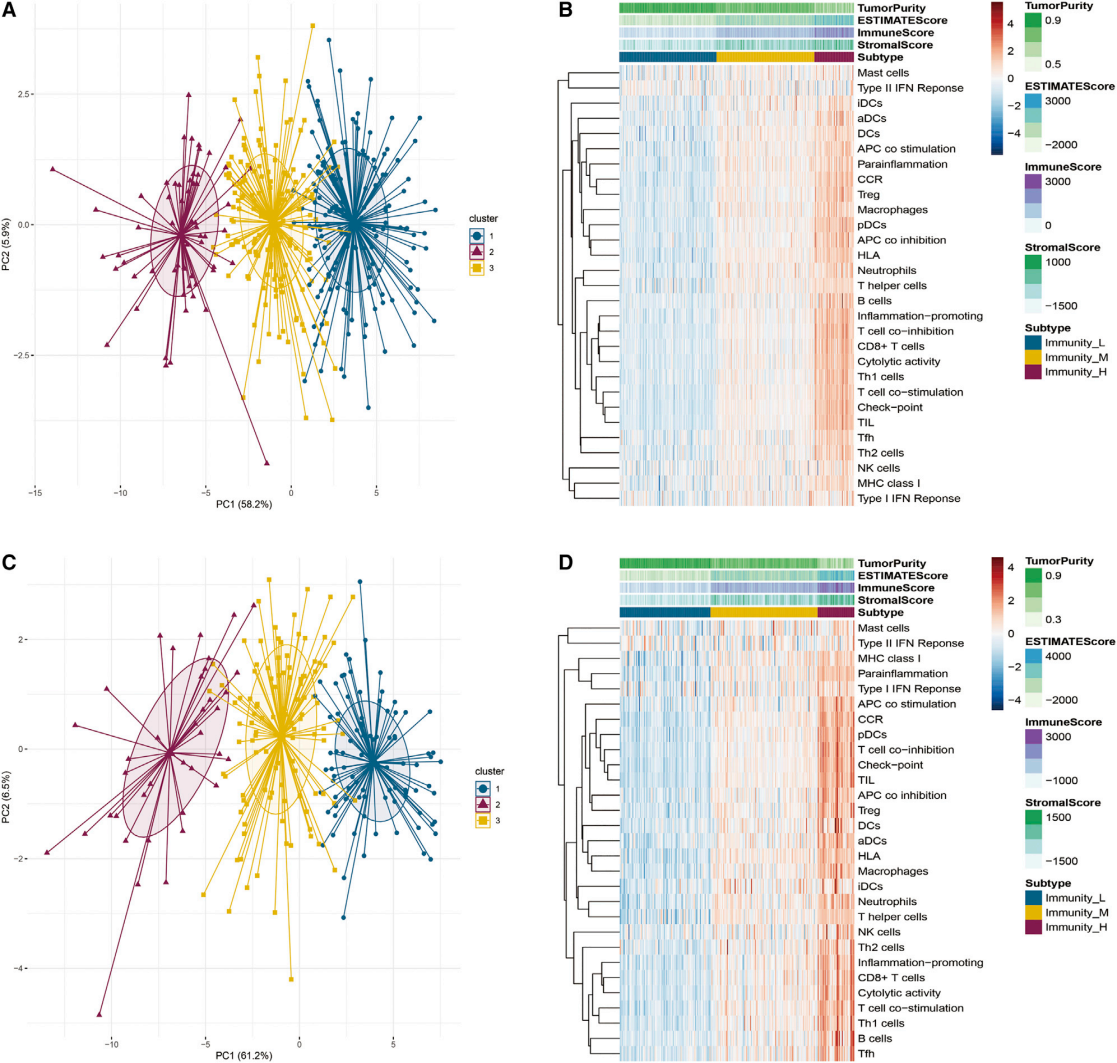
Immune-related pathways profiling identified three HCC clusters (A and B) Based on the different gene expression of 29 pathways, HCC was clustered into three main subtypes: Immunity_H, Immunity_M, and Immunity_L in TCGA. (C and D) HCC was also clustered as Immunity_H, Immunity_M, and Immunity_L in LIRI_JP.

We used single-sample gene set enrichment analysis (ssGSEA) to screen and analyze 29 immune-related pathways. The ssGSEA score obtained represented the activity or infiltration levels of immune cells and pathways in tumor samples. Based on the ssGSEA scores of 29 immune-related pathways, we performed hierarchical clustering on both HCC datasets (The Cancer Genome Atlas [TCGA], Liver Cancer-RIKEN, JP [LIRI_JP]). Interestingly, the clustering results of the analysis in the two datasets were similar, and the patients were divided into three unique immune features (Immunity_H, Immunity_M, and Immunity_L) (Figure 1).

**Figure 2:**
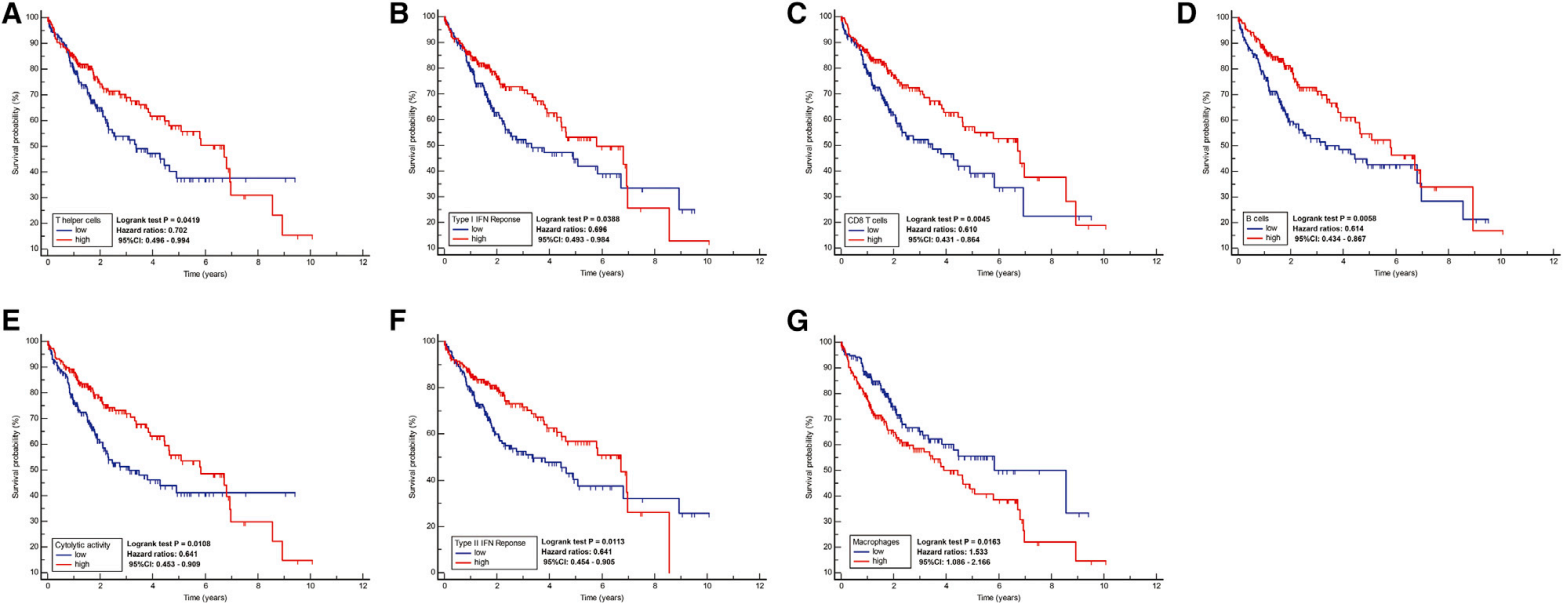
Survival analysis shows that there were 7 immune pathways associated with OS Survival rates and times are shown for the immune-related pathways. (A) T helper cells. (B) Type I IFN Reponse. (C) CD8+ T cells. (D) B cells. (E) Cytolytic activity. (F) Type II IFN reponse. (G) Macrophages.

We screened out 7 significant immune-related pathways (T helper cells, Type I IFN Reponse, CD8+ T cells, B cells, cytolytic activity, type II IFN reponse, macrophages) related to overall survival (OS) of HCC in TCGA and plotted the survival curves. Importantly, we found that patients with high expression levels of these immune-related pathways had better survival prognosis except macrophages (Figure 2).

**Figure 3:**
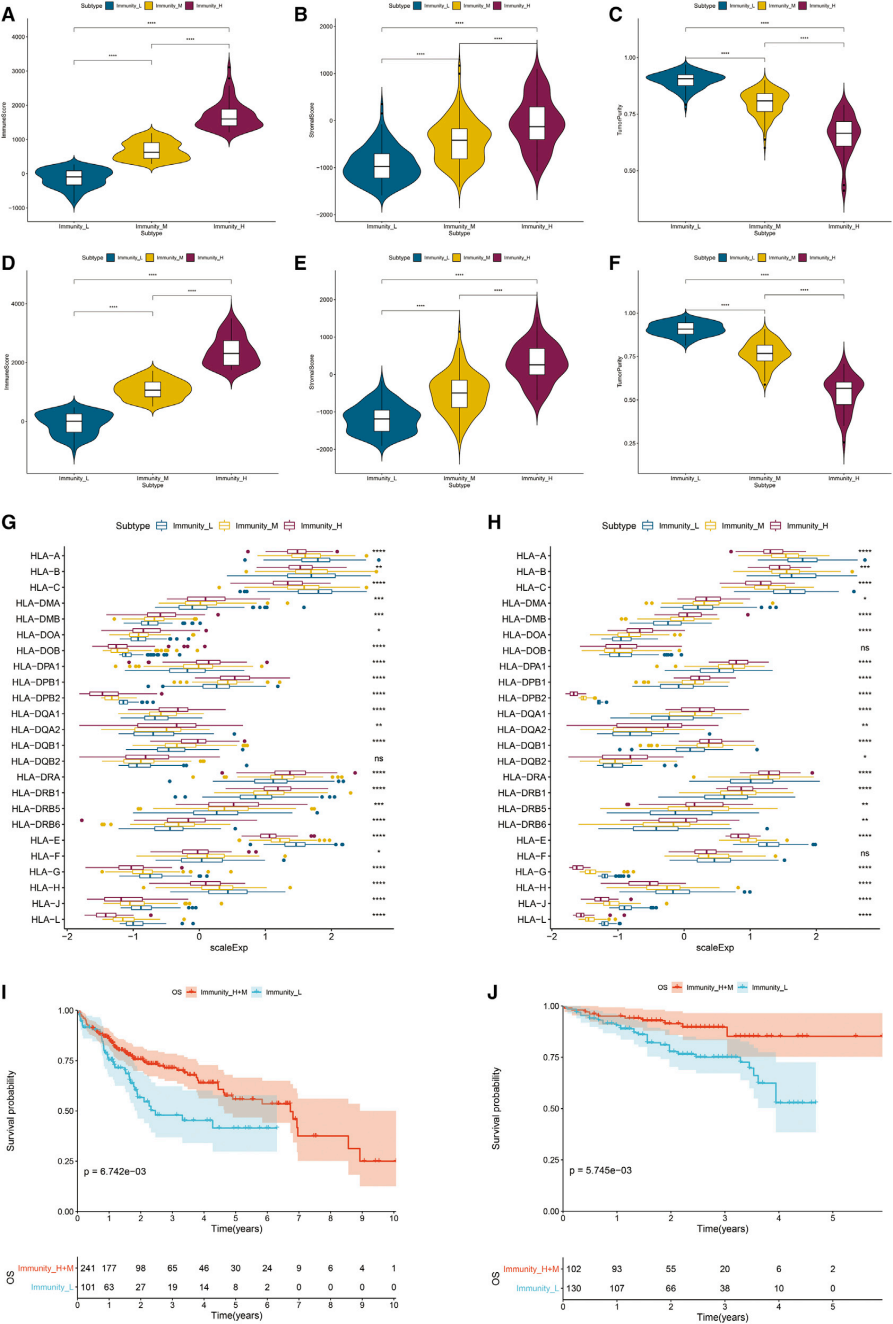
Three HCC subtypes show differential phenotypes (A–C) Comparison of the immune cell infiltration levels, stromal score, and tumor purity between HCC subtypes in TCGA; (D–F) Comparison also in LIRI_JP. (G and H) Comparison of the expression levels of HLA genes between HCC clusters in TCGA and LIRI_JP; Kaplan-Meier Survival analysis indicates that the two clusters had significantly different survival rates both in (I) TCGA (log-rank test p = 6.742e−03) and (J) LIRI_JP (log-rank test p = 5.745e−03). ∗∗∗∗p < 0.001.

Recent studies showed that tumor-infiltrating immune cells played specific roles during cancer development,^11^^,^^12^ interacting with stromal cells in the tumor microenvironment (TME).^13^ Additionally, different cancer types have different immune cells and cytokines.^14^ Numerous factors have been identified as predictors of prognosis and recurrence in patients with HCC, including the size and number of tumors, the type and density of immune cells in tumors, cell differentiation, and the degree of inflammation.^15^ Therefore, we investigated the tumor microenvironment of the three HCC subtypes. According to the violin plots, we found that the immune scores were significantly higher in Immunity_H than those in Immunity_M or Immunity_L in the two datasets (Kruskal-Wallis test, p < 0.001), while the immune scores were lowest in Immunity_L (Figures 3A and 3D). This indicated that the degree of lymphocyte infiltration was significantly higher in Immunity_H (median, 1,598.95 in TCGA; median, 2,314.70 in LIRI_JP) than that in Immunity_L (median, -92.66 in TCGA; median, 5.23 in LIRI_JP). Moreover, when comparing the stromal scores of the three HCC subtypes, the stromal scores increased from Immunity_L to Immunity_H (Immunity_L < Immunity_M < Immunity_H) (Kruskal-Wallis test, p < 0.001) (Figures 3B and 3E). In contrast, tumor purity increased from Immunity_H to Immunity_L (Immunity_H < Immunity_M < Immunity_L) (Kruskal-Wallis test, p < 0.001) (Figures 3C and 3F). Overall, these results indicated that the largest number of immune cells and stromal cells was found in Immunity_H, and Immunity_L contained the largest number of tumor cells.

At the same time, we found that whether in TCGA or LIRI_JP datasets, most human leukocyte antigen (HLA) genes were expressed at higher levels in Immunity_H and significantly lower in Immunity_L (Figures 3G and 3H).

Kaplan-Meier survival analyses showed that different HCC subtypes had different survival probability and survival prognosis. Then, we performed survival analyses of Immunity_H and Immunity_M against Immunity_L in TCGA and LIRI_JP datasets (Figures 3I and 3J). The results showed that Immunity_H and Immunity_M had a better survival probability than Immunity_L. HCC subtypes with high immune activity had better survival prognosis.

### Identification and verification of differential genes related to HCC subtype-specific immunity, pathways, and networks

**Figure 4:**
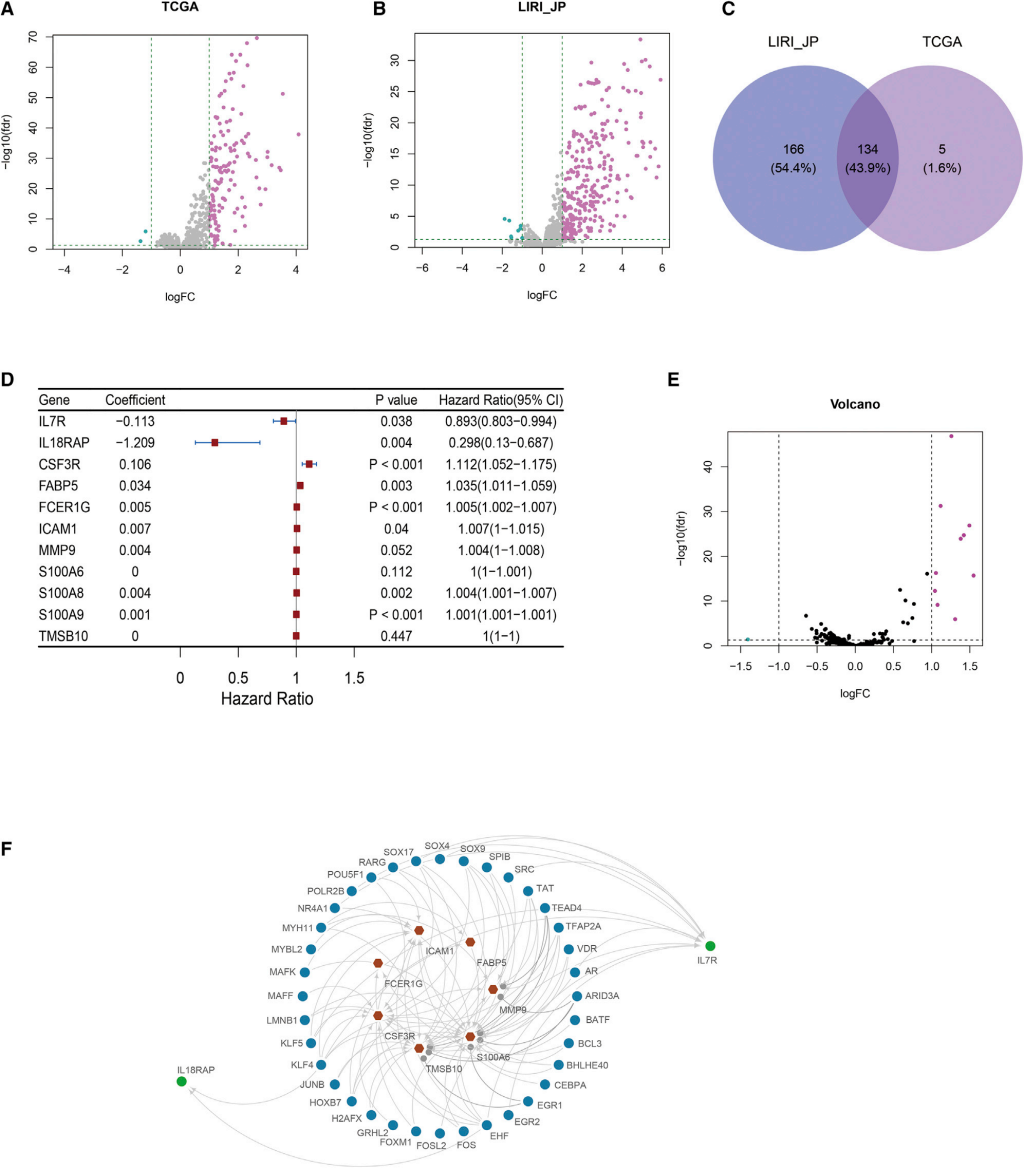
Identification of HCC subtype-specific pathways, gene ontology, and networks (A and B) Volcano plot shows the IRGs that were upregulated or downregulated in TCGA and LIRI_JP. (C) Venn diagram shows that 134 DEIGs were overlapped among TCGA and LIRI_JP. (D) In TCGA, univariate Cox analysis shows there were 11 significant IRGs contributing to OS in HCC. (E) Volcano plot shows differentially expressed TFGs. (F) Correlation analysis of IRGs and TFGs.

We analyzed the differential genes of TCGA and LIRI_JP in HCC immune subtypes (Figures 4A and 4B). There were 139 differentially expressed immune genes (DEIGs) in TCGA and 300 DEIGs in LIRI_JP, and 134 common DEIGs among the two datasets in the Venn diagram (Figure 4C). Based on the univariate Cox regression analysis, we found 11 DEIGs mainly associated with cell adhesion, cell recognition, and signal transduction, of which the hazard ratio (HR) of *IL7R* and *IL18RAP* was less than 1 (*IL18RAP* = 0.298, p = 0.004, 95% confidence interval [CI] = 0.130–0.687; *IL7R* = 0.893, p = 0.038, 95% CI = 0.803–0.994), while the HR of the remaining 9 differential genes was more than 1 (*CSF3R* = 1.112, p < 0.001, 95% CI = 1.052–1.175; *FABP5* = 1.035, p = 0.003, 95% CI = 1.011–1.059; *FCER1G* = 1.005, p < 0.001, 95% CI = 1.002–1.007; *ICAM1* = 1.007, p = 0.040, 95% CI = 1.001–1.015; *MMP9* = 1.004, p = 0.052, 95% CI = 1.000–1.008; *S100A6* = 1.000, p = 0.112, 95% CI = 1.000–1.001; *S100A8* = 1.004, p = 0.002, 95% CI = 1.001–1.007; *S100A9* = 1.001, p < 0.001, 95% CI = 1.001–1.001; *TMSB10* = 1.000, p = 0.447, 95% CI = 1.000–1.000) (Figure 4D), which indicated that *IL7R* and *IL18RAP* were protective genes, and those remaining were harmful genes. In addition, the GSE 14520 dataset and Kaplan-Meier Plotter^16^^,^^17^ were used to cross-validate the two genes, *IL7R* and *IL18RAP*. The results also showed that patients with higher expression of *IL7R* and *IL18RAP* had higher survival (Figure S1). Furthermore, the associations between expression levels of *IL7R* and *IL18RAP* and prognosis were further clarified in 44 randomly selected HCC tissues as investigated by quantitative PCR (qPCR). The expression levels of *IL7R* and *IL18RAP* in surviving individuals were higher than those in dead individuals (Figure S2). These results evidently demonstrated that *IL7R* and *IL18RAP* were downregulated in HCC patients on mRNA, implying the importance of *IL7R* and *IL18RAP* in HCC pathogens. The above results all indicated that *IL7R* and *IL18RAP* might have protective activities.

Many studies have shown that transcription factors are involved in intracellular signal transduction and promoted tumor metastasis and development by releasing a large number of cell growth-promoting factors. We identified the differentially expressed transcription factor genes (TFGs) by plotting volcanic maps in HCC immune subtypes. At the same time, we screened differentially expressed immune-related genes (IRGs) in TCGA by univariate Cox regression analysis. In order to assess the relationship between IRGs and TFGs in HCC, an IRGs-TFGs network was conducted including 9 IRGs and 36 TFGs. The results showed that *IL7R* was regulated by 11 TFGs, including EGR1, EGR2, FOS, JUNB, KLF4, MYH11, NR4A1, RARG, SOX17, SPIB, and VDR. However, the upregulated gene, *IL18RAP*, was regulated by two TFGs, including EGR2 and JUNB (Figure 4F). This phenomenon suggested that *IL7R* and *IL18RAP* might have different mechanisms for regulating immune protection.

### Quantitative analysis of HCC infiltrating immune cells

**Figure 5:**
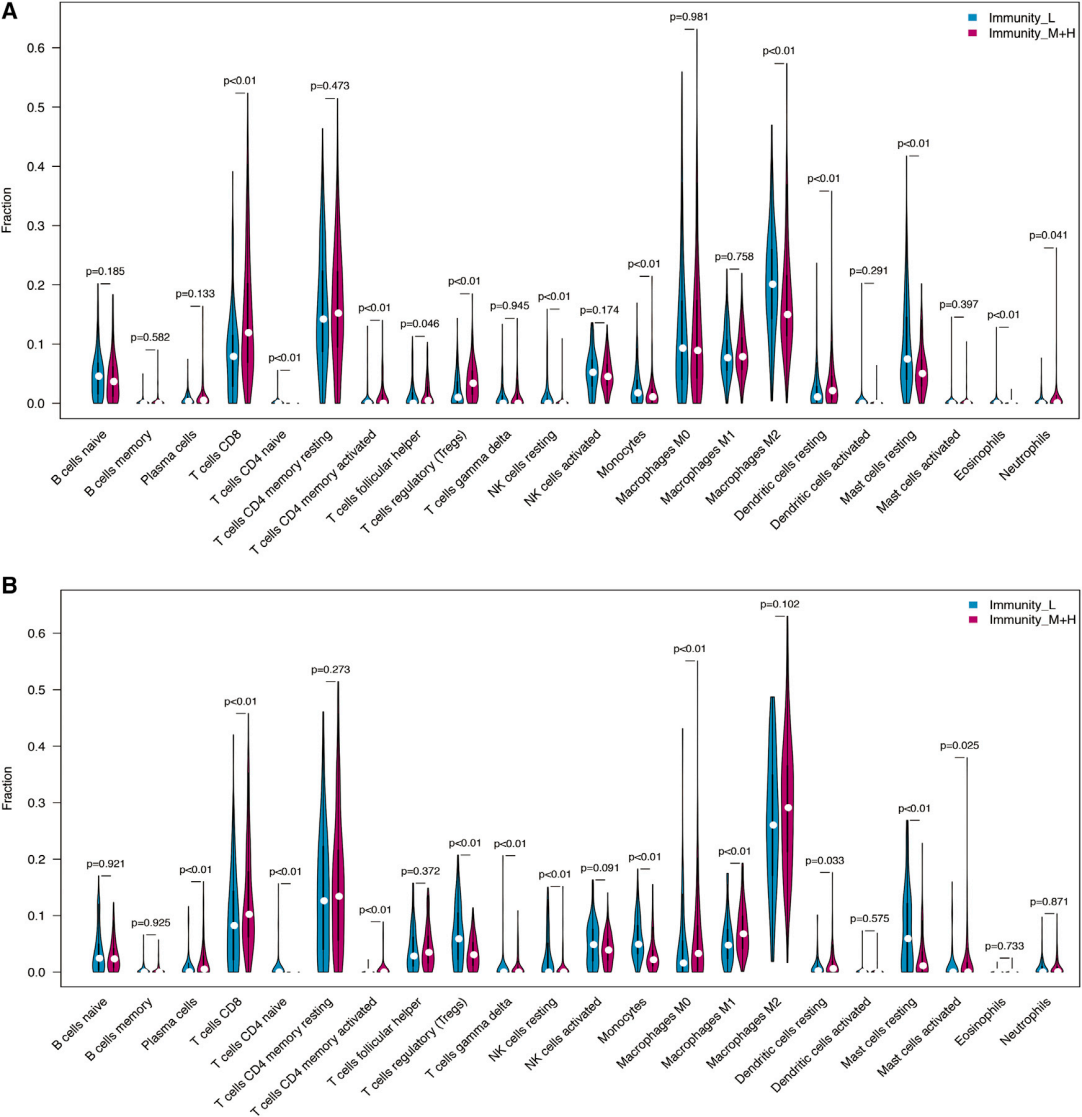
Distribution of immune cells in the immunity_L and immunity_M+H. (A) Clusters in TCGA. (B) Clusters in LIRI_JP.

CIBERSORT was used to calculate immune cell expression of HCC subtypes in TCGA and LIRI_JP datasets. We found that some immune cells were obviously higher in Immunity_H and Immunity_M, such as CD8 T cells, activatived mermory CD4 T cells. In contrast, naive CD4 T cells, monocytes, and resting ast cells were higher in Immunity_L (Wilcoxon test, p < 0.001) (Figure 5)

### Impact of risk score and other factors on survival prognosis of patients with HCC

**Figure 6:**
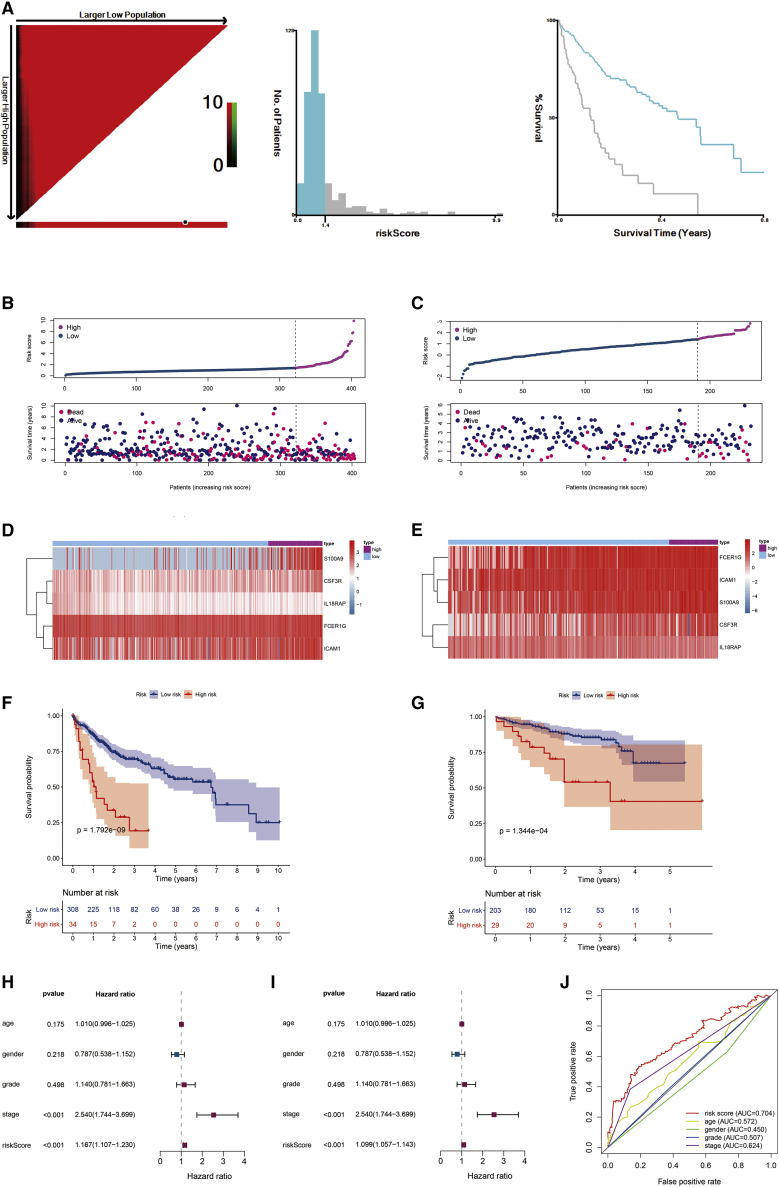
The survival predictor model based on five DEIGs (A) Construction of a risk score through a multi-factor COX model. (B) HCC patients were divided into two groups by the median risk score cutoff point in TCGA: high risk and low risk. (C) According to the same cutoff point, HCC patients were also divided into two groups in LIRI_JP. (D and E) Five DEIGs distinguish two risk groups; Kaplan-Meier survival analysis indicated that the two groups had significantly different survival rates both in (F) TCGA and (G) LIRI_JP. (H) Univariate Cox analysis of risk factors. (I) Multivariate Cox analysis of risk factors. (J) ROC curve of risk factors.

To build a multiple risk scores model, we used multivariate Cox regression analysis and selected 1.4 as the risk score cutoff point based on a risk scores model that divided HCC patients into two groups: high risk and low risk in TCGA and LIRI_JP datasets (Figures 6A–6C). By LASSO regression analysis, five differentially expressed rhythm genes (DERGs) (*S100A9*, *CSF3R*, *IL18RAP*, *FCER1G* and *ICAM1*) were screened out. Moreover, we quantified the enrichment levels of the five DERGs in two groups by ssGSEA, and the results showed totally different levels of those DERGs in the heatmap (Figures 6D and 6E). Also, Kaplan-Meier analysis indicated that patients with high risk had shorter survival times and lower survival rates in TCGA (log-rank test p = 1.792e−09) and LIRI_JP (log-rank test p = 1.344e−04) (Figures 6F and 6G).

Meanwhile, we performed univariate and multivariate Cox analyses of some variables (age, sex, grade, stage, and risk score) in TCGA. In univariate Cox analysis, tumor stage (HR = 2.540, p < 0.001, 95% CI = 1.744–3.699) and risk score (HR = 1.167, p < 0.001, 95% CI = 1.107–1.230) were associated with the survival rates (Figure 6H). Furthermore, the multivariate Cox analysis indicated that tumor stage (HR = 2.540, p < 0.001, 95% CI = 1.744–3.699) and risk score (HR = 1.099, p < 0.001, 95% CI = 1.057–1.143) were correlated with OS (Figure 6I). According to comparison of the AUC (area under curve) of the receiver operating characteristic (ROC) curve, we also identified that risk score (AUC = 0.704) predicted mortality more accurately than did the other HCC prognostic factors: age (AUC = 0.572), sex (AUC = 0.450), grade (AUC = 0.507), stage (AUC = 0.624) (Figure 6J).

## Discussion

As the biggest immune organ, the liver plays an important role in immune responses.^18^ C-reactive protein (CRP) is an acute phase reactant protein considered as a diagnostic indicator of early inflammation, which is synthesized by the liver.^19^ Furthermore, the liver is engaged in inflammation, and elevated inflammation will cause liver damage.^20^ Immune system disorders are associated with lymphocyte infiltration of the liver.^21^^,^^22^ Therefore, the liver is essential to the regulation of immune defense.^23^ Thus, immune pathways in the liver may provide more refined prognostic prediction for liver diseases.

In this study, HCC was divided into three major subtypes, Immunity_H, Immunity_M, and Immunity_L, according to immune pathways. The three groups showed significant differences in anti-tumor immune activity, immune cell infiltration, and immune pathways, such as ESTIMATE score, immune score, stromal score, innate immunity, and adaptive immunity. The immune system of the liver responds to diverse pathogens mainly in two fundamental pathways: recognizing and destroying pathogens or remembering specific pathogens and efficient targeted killing.^24^^,^^25^ The theory is consistent with the results of our study, indicating that the Immunity_H subtype with a higher expression level of innate immunity and adaptive immunity likely had a better survival prognosis (Figures 3I and 3J). Moreover, the three subtypes showed a significant difference on the composition and proportion of immune cells. Additionally, cytokines also correlate with the diagnosis and prognosis for HCC as biomarkers and regulators of tumor proliferation, invasion, migration, and apoptosis,^26^, which were induced by immune cells, including IFN and TNF-α.^27^^,^^28^ Traditional Chinese medicine (TCM) serves an antitumor role by regulating the expression of cytokines, such as β-elemene, to improve the OS rate.^29^ In conclusion, the immune pathways discussed above were associated with the development and prognosis of HCC.

Immune cells in the tumor microenvironment are complex and diverse, including T lymphocytes (70%−80%), B lymphocytes (10%−20%), macrophages (5%−10%), and NK cells (<5%), and dendritic cells (1%–2%).^30^ Additionally, regulatory T lymphocytes (Tregs) and tumor-associated macrophages (TAMs) contribute to tumor escape with immune suppressive activity and inhibit anti-tumor responses. Immune cells infiltrating tumors mediate the tumor immune microenvironment.^31^ These immune cells are all associated with the prognosis of HCC. A large number of studies have shown that the density of tumor-infiltrating lymphocytes (TILs) was positively correlated with survival prognosis in various cancers.^32^ Moreover, HCC immunotherapy works on these immune cells. Based on the different expression levels of immune cells, we classified HCC subtypes and filtered out HCC patients who could benefit from immunotherapy.^33^

Previous studies have shown that the expression levels of different IRGs also had a certain effect on the survival prognosis of patients with HCC.^34^^,^^35^ Among the five DEIGs, *S100A9* is involved in cell growth, differentiation, and apoptosis and promotes tumor metastasis.^36^ *IL18RAP* effectively inhibits the growth of HCC cells by inhibiting angiogenesis and apoptosis signal transduction involving caspase-3, which fully demonstrates the antitumor effect of *IL18RAP*.^37^ In our study, we divided HCC patients into two groups based on the five DEIC-based classification in TCGA and LIRI_JP, and verified the accuracy and reliability of the classification. Then, we found that the two groups showed different survival rates in TCGA and LIRI_JP. Furthermore, the risk score was the best predictor when compared with the other risk factors, which was the focus of our research. The combination of these risk factors may lead to a more accurate prediction of HCC prognosis. Moreover, we found that the expression levels of *IL7R* and *IL18RAP* in HCC were higher in surviving individuals than in individuals who died through the verification of clinical samples and multiple datasets, indicating that *IL7R* and *IL18RAP* were protective genes in HCC. Therefore, the DEIG-based survival predictor model has shown a favorable influence on survival prediction, which might contribute to treatment decision making.

However, the study has some limitations. First, previous studies showed that tumor immunity was closely associated with tumor metabolism.^38^ However, our study mainly focused on the relationship between the IRGs and the OS of HCC. The conjoint analysis of IRGs and metabolism-related genes (MRGs) would be more propitious to investigate the prognosis of HCC. Second, only two databases (TCGA, LIRI_LP) were available for immunogenomic classification of HCC at present, so we need more databases in the future to improve the accuracy of the classification. Third, in our study, accessible clinical samples were limited. It would be even more clinically valuable if we could find tumor biomarkers detected in blood samples that are more readily available.

In summary, our study identified three immune-based classifiers closely related with prognosis in HCC. Furthermore, the DEIG-based survival predictor model could accurately predict the OS of HCC patients, which may facilitate individual immunotherapy in HCC.

## Materials and methods

### Patient datasets

The gene expression data and clinical information of HCC patients were extracted from TCGA (https://portal.gdc.cancer.gov/) and Liver Cancer-RIKEN, JP (LIRI_JP) from the International Cancer Genome Consortium (ICGC) (https://dcc.icgc.org/). Kaplan-Meier Plotter (http://kmplot.com/analysis/) and the GEO: GSE14520 dataset consisting of 228 HCC samples downloaded from Gene Expression Omnibus (https://www.ncbi.nlm.nih.gov/geo/) were used to validate the two most important protective genes, *IL18RAP* and *IL7R*, for prognosis of HCC patients.

### Clustering

First, data from TCGA and LIRI_JP were subjected to GSEA based on 29 immune-related pathways. For each HCC-independent dataset, a ssGSEA score was used to calculate the enrichment levels of the 29 immune pathways in each HCC sample. According to the ssGSEA score calculated, hierarchical clustering was conducted for HCC.

### Correlation between immune pathways and prognosis

A single sample of ssGSEA was used to analyze 29 immune pathways, and the “survival” package of R software was used to screen prognostic immune pathways.

### Evaluation of immune cell infiltration level, tumor purity, and stromal content in the clusters

ESTIMATE^39^ was used to calculate the score of the immune microenvironment, such as the infiltration level of immune cells (immune score), tumor purity, and stromal content (stromal score). The violin plot was present based on the score in Immunity_H, Immunity_M, and Immunity_L.

### Comparison of the proportions of infiltrating immune cells between immunity subtypes

We used CIBERSORT (https://cibersort.stanford.edu/) to estimate the proportions of 22 infiltrating human immune cells in TCGA and LIRI_JP of each sample. The violin plot was presented based on the different proportion of immune cells.

### GSEA

We performed GSEA of TCGA and LIRI_JP datasets by GSEA (R GSVA package).^40^ This analysis respectively identified the immune related genes that were upregulated or downregulated in HCC. We created a Venn diagram to select the common immune genes in both datasets.

### Identification of differential genes related to HCC subtype-specific immunity and networks

The IRGs that were upregulated or downregulated in HCC patients were shown in a volcano plot. A Venn diagram was plotted based on the difference in genes of the TCGA and LIRI_JP datasets, and we identified the common IRGs in the two datasets. In addition, differentially expressed genes were identified in the database by univariate regression analysis. Through the correlation analysis of the differential IRGs and differential TFGs, we established gene-gene interaction networks, and the hub genes were defined as TFGs.

### Risk scores for HCC patients

Multivariate Cox regression analysis was conducted to establish a risk score of each patient. The optimal cutoff value was screened out by X-tile, which classified the patients into a high-risk group and a low-risk group. Based on the risk score, we drew a risk score scatterplot. The LASSO regression method was used to screen DEIGs,^41^ and the enrichment levels were quantified by ssGESA. At the same time, we used the survminer package to draw a Kaplan-Meier curves analysis diagram. Survival curves were used to show the differences in survival time and survival probability between high-risk and low-risk patients.

### RNA extraction and qPCR

A total of 44 HCC tissues samples (stored at −80°C) were obtained from 44 HCC patients undergoing liver cancer surgery at The First Affiliated Hospital of Wenzhou Medical University after having given their informed consent. In addition, the clinical data of HCC patients (n = 44), including age, sex, stage, HBV infection, tumor range, diabetes, and relapse, were collected (Table S1). The research protocol of the study was approved by the Ethics Committee of The First Affiliated Hospital of Wenzhou Medical University (2019-070). Samples of 50 mg of tissue were washed with PBS and transferred into a 1.5-mL tube containing 0.5 mL of RNAiso Plus (Takara, Japan) as well as two grinding beads, homogenizing completely with tissue grinders. The mixture was centrifuged at 12,000 × *g* for 5 min at 4°C. The supernatant was transferred to a new tube, then total RNA was extracted according to the manufacturer’s protocol. We used NanoDrop and an Agilent 2100 bioanalyzer (Thermo Fisher Scientific, MA, USA) to determine the concentration of extracted total RNA. cDNA was obtained by reverse transcription using a reverse transcription kit (Hiscrip II Q RT SuperMix for qPCR) according to the manufacturer’s protocol. Quantitative real-time polymerase chain reaction amplification was performed with SYBR Green PCR master mix (Takara, Japan) according to the manufacturer’s protocol. Expression of transcripts was assessed by the following primers: *IL7R*, forward, 5′-TAATGCACGATGTAGCTTACCG-3′, reverse, 5′-CTTTCTCTGCAGGAGTGTCAG-3′; *IL18RAP*, forward, 5′-C GTATCCTATGCAAAATGGAGC-3′, reverse, 5′-CAAGCAAACACAGGCTATAT CC-3′; *FCER1G*, forward, 5′-CAGTGGTCTTGCTCTTACTCC-3′, reverse, 5′-ATG GCAT CCAGGATATAGCAG-3′.

### Survival analysis

We compared survival probabilities of HCC patients based on HCC subtypes and the expression levels of the identified genes. We used the “survival” package for survival analysis with the available survival data in TCGA and LIRI_JP datasets and plotted Kaplan-Meier curves to represent the difference in survival time. We performed univariate and multivariate Cox analysis to identify significant prognostic predictors associated with OS, such as age, sex, grade, stage, risk score, and other variables of HCC patients, and plotted forest maps. The AUC of ROC curves represented the predictive accuracy. In addition, p values <0.05 were considered significant.

## Supplemental information

Document S1. Figures S1 and S2 and Table S1

Document S2. Article plus supplemental information
