# Supplementary material for: An immunogenomic signature for molecular classification in hepatocellular carcinoma
Source: Mol Ther Nucleic Acids. 2021 Jul 2;25:105–15. doi: 10.1016/j.omtn.2021.06.024 (PMC8332372; doi:10.1016/j.omtn.2021.06.024)
Supplement: Document S1. Figures S1 and S2 and Table S1 [file mmc1.pdf]

OMTN, Volume *25*

Supplemental information

An immunogenomic signature for molecular classification in hepatocellular carcinoma

Weiwei Zhuang, Hongwei Sun, Shanshan Zhang, Yilin Zhou, Wanqing Weng, Boda Wu, Tingbo Ye, Weiguo Huang, Zhuo Lin, Liang Shi, and Keqing Shi

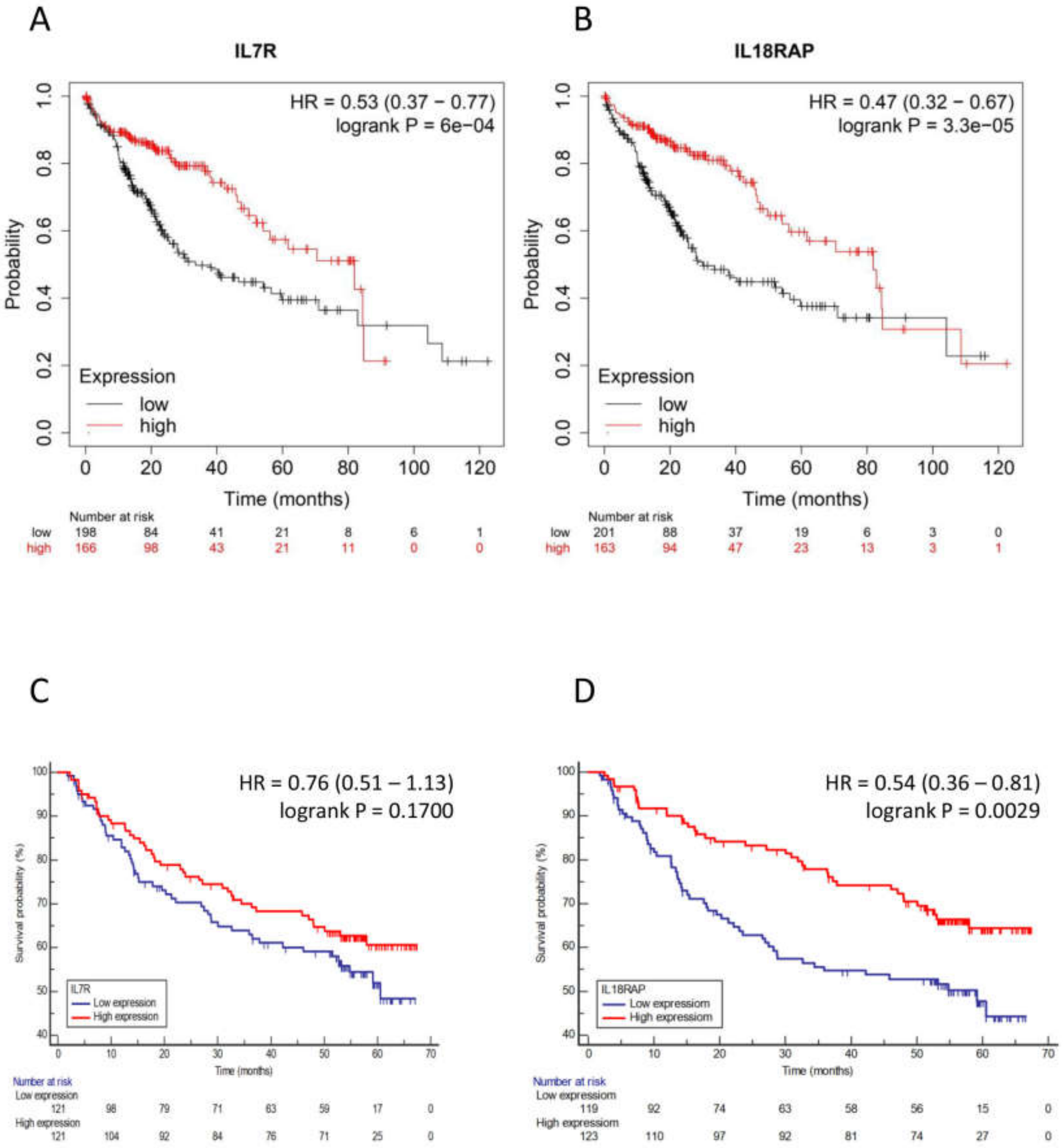

Supplementary Figure 1. Cross-validation of IL7R and IL18RAP in prognosis of hepatocellular carcinoma using Kaplan-Meier Plotter and GEO (GSE 14520) databases.

(A) IL7R in Kaplan-Meier Plotter databases; (B) IL18RAP in Kaplan-Meier Plotter databases; (C) IL7R in GSE 14520; (D) IL18RAP in GSE 14520

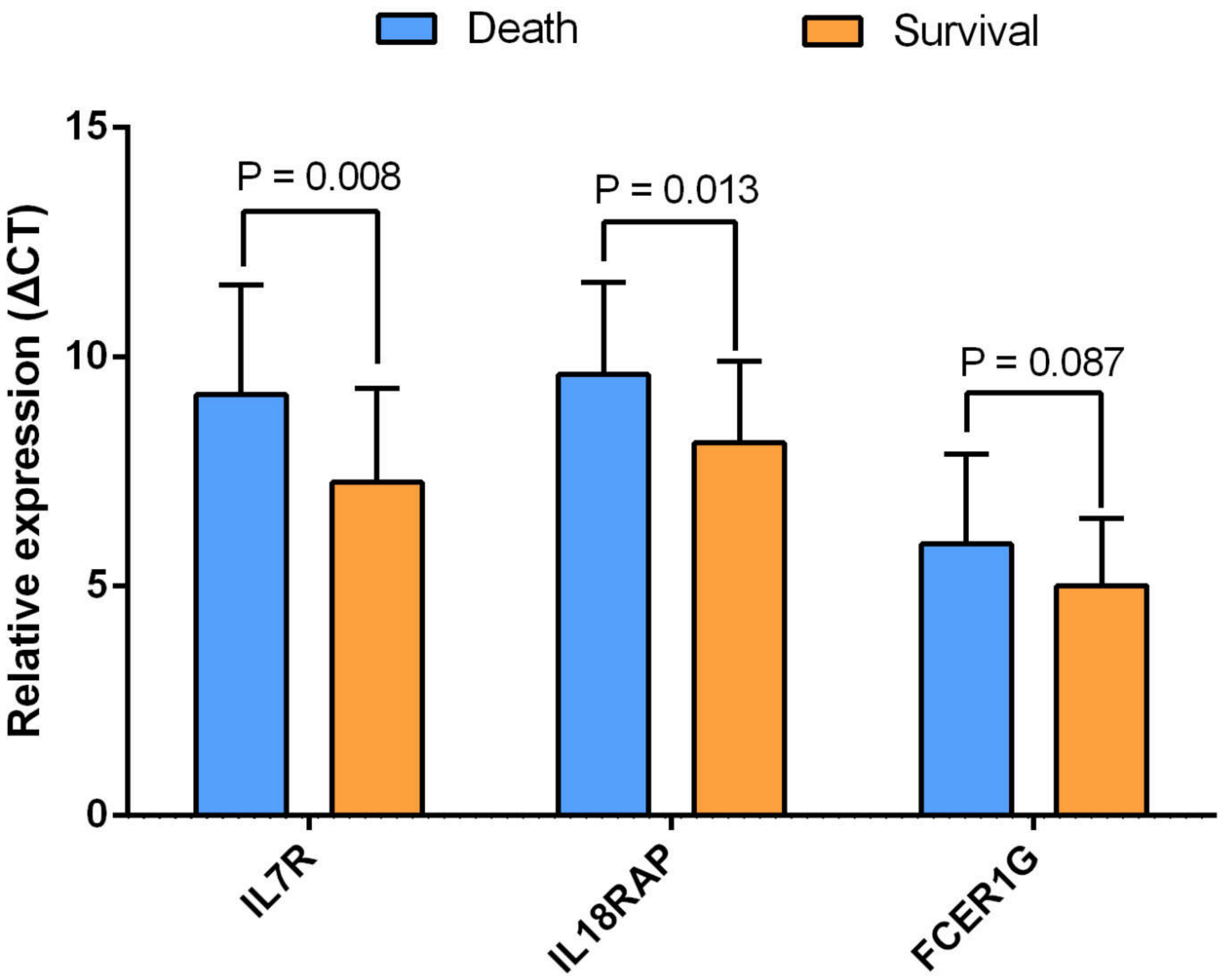

Supplementary Figure 2. The expression levels of IL7R and IL18RAP in randomly selected hepatocellular carcinoma samples.

Table S1. The clinicopathological characteristics of the three cohorts

Variables	Subgroups	Development set (N=44)
**Age**		
	<60	28
	≧60	16
**Gender**		
	Male	37
	Female	7
**HBV infection**		
	Positive	31
	Negative	13
**HCV infection**		
	Positive	0
	Negative	44
**AFP**		
	<300ng/mL	30
	⩾300ng/mL	14
**CA19-9**		
	<20U/ml	26
	⩾20U/ml	15
	Unknow	3
**Total bilirubin**		
	<21umol/L	36
	⩾21umol/L	8
**ALT**		
	>50U/L	16
	⩽50U/L	28
**AST**		
	>50U/L	14
	⩽50U/L	30
**GGT**		
	>50U/L	22
	⩽50U/L	22
**Degree of tumor differentiation**		
	Poorly	4
	Poorly moderately	9
	Moderately	19
	Moderately well	2
	Well	3
	Unknow	7
**TNM stage**		
	Stage Ⅰ	14
	Stage Ⅱ	8
	Stage Ⅲa	2

	Stage III b	1
	Stage III c	6
	StageIV	2
	Unknow	11
**Tumor range**		
	I	14
	II	10
	III	3
	IV	5
	Unknow	12
**Lymphatic metastasis**		
	Yes	10
	No	24
	Unknow	10
**Remote metastasis**		
	Yes	2
	No	30
	Unknow	12
**Liver cirrhosis**		
	Yes	28
	No	16
**Diabetes**		
	Yes	8
	No	36
**Relapse**		
	Yes	28
	No	16
**Survival status**		
	Dead	19
	Living	25
